# The Financial Impact of Hypofractionated Radiation for Localized Prostate Cancer in the United States

**DOI:** 10.1155/2019/8170428

**Published:** 2019-01-02

**Authors:** Assaf Moore, Ido Stav, Robert B. Den, Noa Gordon, Michal Sarfaty, Victoria Neiman, Eli Rosenbaum, Daniel A. Goldstein

**Affiliations:** ^1^Institute of Oncology, Davidoff Cancer Center, Rabin Medical Center, Petah Tikva, Israel; ^2^Sackler Faculty of Medicine, Tel Aviv University, Tel Aviv, Israel; ^3^Department of Radiation Oncology, Jefferson University, Philadelphia, PA, USA; ^4^Department of Health Policy and Management, University of North Carolina, Chapel Hill, USA

## Abstract

**Background:**

Until recently, dose intensified radiotherapy was the standard radiation method for localized prostate cancer. Multiple studies have demonstrated similar efficacy and tolerability with moderate hypofractionation. In recent years there has been an increasing focus placed on understanding the cost and value of cancer care. In this study we aimed to assess the economic impact of moderate hypofractionation for payers in the United States.

**Methods:**

We performed a population-based analysis of the total cost of external beam radiotherapy (EBRT) for localized prostate cancer in the US annually. The national annual target population of patients treated with definitive EBRT was calculated using the Surveillance, Epidemiology, and End Results (SEER) database. Treatment costs for various fractionation schemes were based on billing codes and 2018 pricing by the Centers for Medicare and Medicaid Services (CMS).

**Results:**

We estimate that 27,146 patients with localized prostate cancer are treated with EBRT annually in the US. The cost of standard fractionation in 45 or 39 fractions is US$ 26,782 and 23,625 per patient, respectively. With moderate hypofractionation in 28 or 20 fractions, the cost is US$ 17,793 and 13,402 per patient, respectively. The use of moderate hypofractionation would lead to 25-50% annual savings US$158,315,472-US$363,213,480 in the US.

**Conclusions:**

Moderate hypofractionation may have the potential to save approximately US$0.16-0.36 billion annually, likely without impacting survival or tolerability. This may lead to lower personal financial toxicity. It would be reasonable for public and private payers to consider which type of radiation is most suited to reimbursement.

## 1. Introduction

Prostate cancer is the third most common cancer diagnosed in the US and represents 9.6% of all new cancer cases [1, 2]. In 2018, the American Cancer Society estimates that 164,690 patients will be diagnosed with prostate cancer, and 29,430 will succumb to their disease [2]. Seventy-nine percent of prostate cancer patients are diagnosed with localized disease, defined as a tumor that is confined to the primary site [1, 2]. In general, there are three treatment options: surgical resection, radiation with or without hormonal therapy, and finally active surveillance when appropriate. The choice of these options is largely driven by disease risk status, age, and patient preferences. Localized prostate cancer is heterogeneous, and while some men may have a more aggressive course of disease, others do not.

Dose escalation studies in localized prostate cancer have demonstrated improved outcomes compared to lower doses [3–7]. In light of these findings, the standard of care is to use a dose of 75.6-81 Gy depending on the risk stratification group [8]. High biologically equivalent doses can be achieved with various fractionation schemes. One such way is moderate hypofractionation. The linear quadratic model uses the *α*/*β* ratio to describe the varying effect of fraction size on cell survival. The higher the ratio, the more “radiosensitive” the tumor. As prostate cancer is considered to have a low *α*/*β* ratio, higher doses per fraction could potentially improve prostate cancer cell killing. Several observational reports of moderate hypofractionation demonstrated an acceptable safety profile [9–11]. Following these reports were a series of randomized controlled trials analyzing the safety and efficacy of hypofractionated radiation compared to traditionally fractionated radiation for patients with localized prostate cancer. These trials varied in design as well as having differing inclusion and exclusion criteria. While most trials included mainly low and intermediate risk patients [12–16], some have focused on high risk [17–19]. In essence these studies found an equivalent level of efficacy and safety when using hypofractionated radiation [12–15] (Table 1). Moderate hypofractionation was not found to be superior to traditional fractionation in terms of survival, [17–19] but was found to be noninferior to conventional fractionation for biochemical control with similar overall and cancer specific survival [12–14] (Table 1).

Despite concerns for an increase in late toxicity with higher doses per fraction, most trials have not found a statistically significant difference [13–15, 17, 18]. One exception is RTOG 0415, where men assigned to hypofractionation had significantly more late grade 2 GI and GU toxicity (HR 1.59, p=0.005 and HR 1.31, p=0.009, respectively) [12]. Another exception is the HYPRO trial that used a 3.4 Gy dose per fraction in the hypofractionated arm and found that the cumulative incidence of grade 3 or worse late GU toxicity was significantly higher (19% versus 12.9%, p=0.021) [19]. However, the most relevant index regarding toxicity is patient-reported outcomes. These were found to be equivalent as seen in the 5-year patient-reported outcomes of the CHHiP trial [13, 14]. Another report found no statistically significant difference in long-term quality of life outcomes between conventional and hypofractionated treatment [20].

In light of these accumulating data, the recent guideline update by ASTRO, ASCO, and AUA has determined that moderate hypofractionation should be offered to low risk patients who decline active surveillance, to men with intermediate risk prostate cancer with or without radiation to the seminal vesicles, and to men with high risk prostate cancer receiving EBRT to the prostate only [21].

In recent years there has been an increasing focus placed on understanding the cost and value of cancer care. This has led to the development of various frameworks that aim to understand value [22, 23]. However, the focus has predominantly been placed on the value of pharmaceutical interventions. There are however widespread opportunities for improving value in other fields such as surgery, radiation, and end-of-life care. Between 2010 and 2012, radiotherapy was the primary treatment among 23% and 33-36% of patients under 64 and over 64 years diagnosed with prostate cancer, respectively [1].

The objective of this study was to assess the difference in cost from the payers' perspective of the USA if all patients currently treated with traditional radiation received moderately hypofractionated radiation.

## 2. Methods

### 2.1. Methodological Overview

We performed a population-based budget impact analysis according to the guidelines set forth by the International Society of Pharmacoeconomics and Outcomes Research [24]. We performed the analysis from the payers' perspective in the United States. The budget impact model was developed using Matlab version R2016b (MathWorks, Inc.).

### 2.2. Target Population

We used the Surveillance, Epidemiology, and End Results (SEER) database to estimate the population of patients treated annually with EBRT. We performed a frequency analysis on treatment with EBRT for localized prostate cancer in the latest year summarized in the SEER cancer statistics, 2014. We excluded all patients who were treated with a combination of EBRT and brachytherapy, patients who refused EBRT, patients for whom radiation modality was unknown, and cases in which it is unknown whether EBRT was eventually administered. We then estimated the total number of patients treated in the USA by extrapolation, based on the fact that the SEER database covers approximately 28% of the US population. We therefore multiplied the results by 3.57.

### 2.3. Radiation Treatment Cost Estimates

In order to estimate treatment costs for various fractionation schemes we used billing codes by the Current Procedural Terminology, 4^th^ Edition (CPT®) 2018 pricing by the Centers for Medicare and Medicaid Services (CMS). The reimbursement rates specified are by National Payment Amount, and not by specific locality. We performed multiple analyses to assess the payers cost when different fractionation regimens are used nationwide. The billing codes included and price of various fractionation schemes are presented in Table 2. We regarded all patients treated annually with EBRT as if all were treated with the same fractionation scheme. We then calculated the total annual cost of treatment and then the annual saving for various moderate hypofractionation schedules. As most trials reported equal tolerability, we included EBRT associated cost only with no consideration of adverse event management.

### 2.4. Sensitivity Analysis

A 10% range was applied for all parameters of the model. We performed a univariate sensitivity analysis to assess which parameters had the greatest impact on cost savings. A probabilistic sensitivity analysis was performed using a Monte Carlo Simulation. The model was run 100,000 times, using the parameters included.

## 3. Results

**Figure 1:**
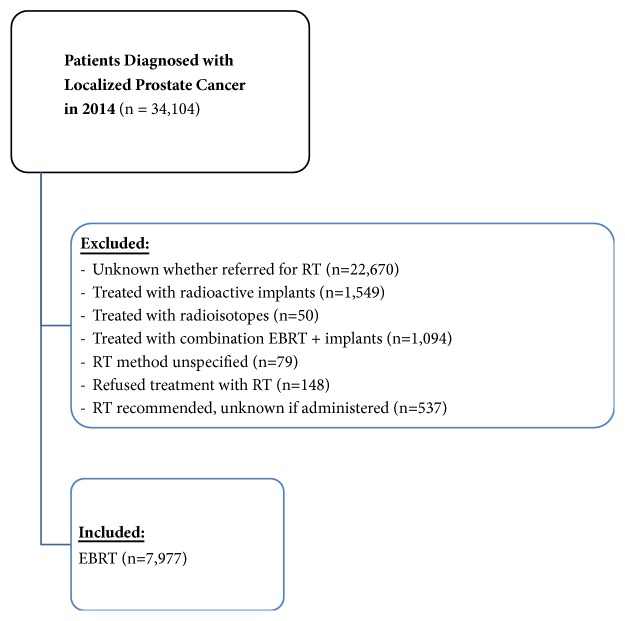
**Target Population.** Data extraction from the Surveillance, Epidemiology, and End Results (SEER) database.

Our frequency analysis of the SEER database found that 34,104 patients were diagnosed with localized prostate cancer in 2014. We excluded 22,670 patients for whom EBRT status was unknown. Of patients referred for radiotherapy, we excluded 373 patients treated with adjuvant EBRT, 1549 patients treated with radioactive implants, 1094 patients treated with a combination of EBRT and radioactive implants, 79 patients for whom the radiation method was unknown, 148 patients who refused treatment, and 537 patients who were recommended to receive radiation therapy but it is unknown whether it was administered. The final analysis included 7,604 patients treated with definitive EBRT as a single modality (Figure 1). As the SEER database covers 28% of the population, we multiplied 7,604 by 3.57 in order to estimate that approximately 27,146 patients would be treated annually with radiation in the USA.

The cost of various fractionation schemes is summarized in Table 1 and ranges between US$ 26,782 for 45 fractions and US$ 13,402 for 20 fractions. The saving per patient ranges between 25% (when comparing a 39- and 28-fraction schedule) and up to 50% (when comparing 45 and 20 fractions).

**Figure 2:**
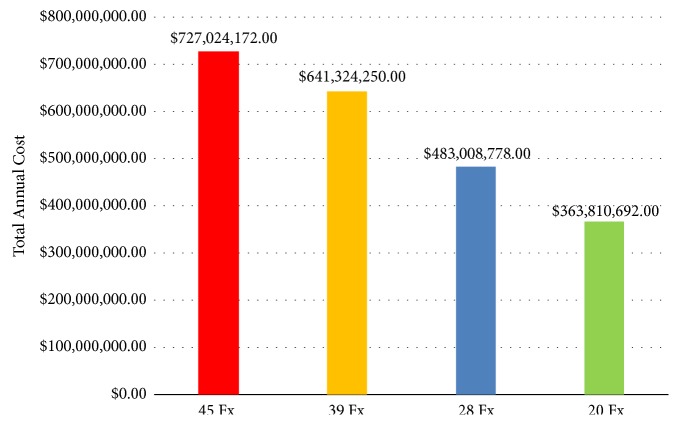
Total annual cost by fractionation scheme.

In our analysis, the annual cost of standard fractionated EBRT is US$727,024,172 and US$641,324,250 for 45 and 39 fractions, respectively. With moderate hypofractionation, the annual cost is US$483,008,778 and US$363,810,692 for 28 and 20 fractions, respectively (Figure 2). Adopting moderate hypofractionation as a new standard of care could lead to a national annual saving of approximately US$158,315,472-US$363,213,480.

**Figure 3:**
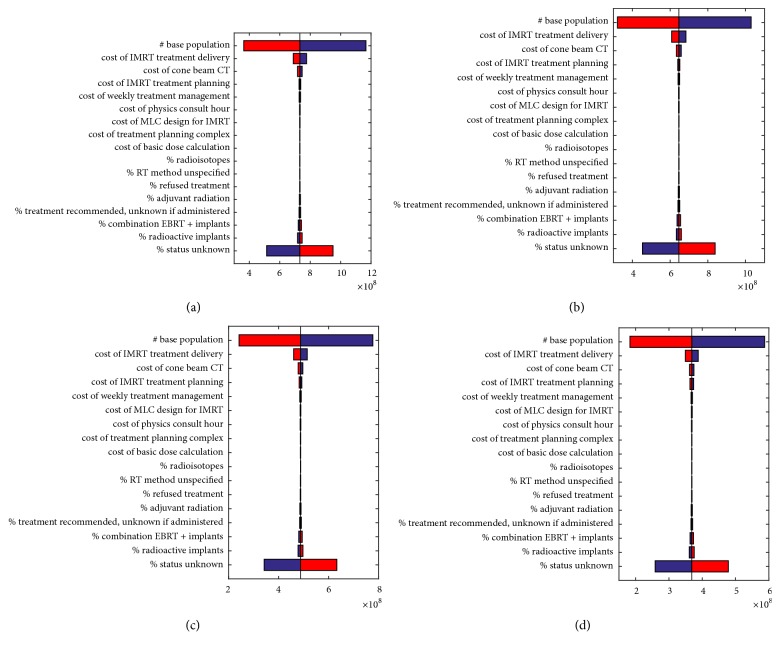
**Univariate sensitivity analysis for various parameters' impact on total cost of EBRT by fractionation scheme.** (a) 45 fractions; (b) 39 fractions; (c) 28 fractions; (d) 20 fractions.

**Figure 4:**
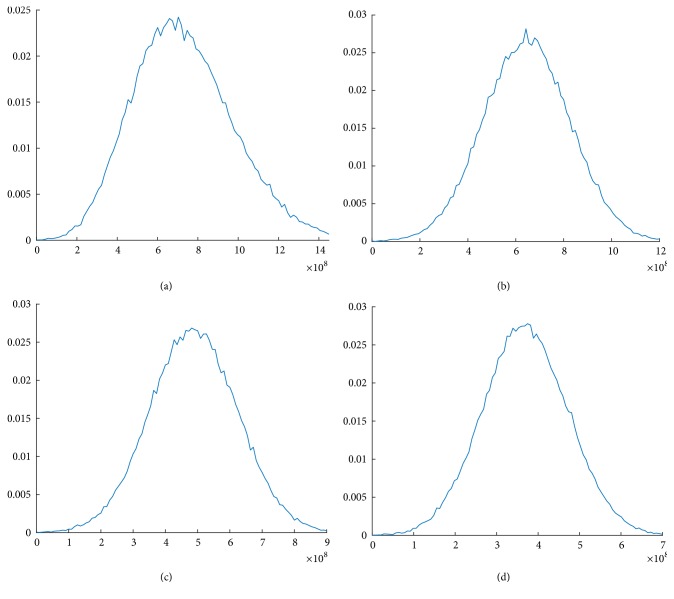
**A probabilistic sensitivity analysis using population and treatment parameters by fractionation scheme.** (a) 45 fractions; (b) 39 fractions; (c) 28 fractions; (d) 20 fractions.

The univariate sensitivity analysis (Figure 3) demonstrates that the model variables with the greatest potential impact on the differences in annual cost of each fractionation scheme are the target population size and the cost of IMRT treatment delivery (i.e., the number of fractions). The results of the probabilistic sensitivity analyses are presented in Figure 4. They demonstrate different probabilities of different levels of budget impact based on the inputs to our model.

## 4. Discussion

We performed an estimation of the impact of moderate hypofractionation on the annual cost of radiation therapy for localized prostate cancer. We found that the annual cost could be decreased by 25-50%. This reduction is mostly attributed to fewer fractions per treatment course. The relevance of this analysis is dependent on the clinical equivalency between standard fractionation and moderate hypofractionation, which has been proven in several randomized trials [12–15].

In an era of numerous medical and technological innovations, treatment costs are rising. High precision radiation techniques are associated with significant expense and are now incorporated into the treatment algorithm of multiple tumors and clinical scenarios. One study has assessed that over one decade the number of patients receiving radiation therapy during their initial treatment course is expected to increase by 22% [25]. As the numbers of linear accelerators and radiation oncologists are finite, it is clear that cutting treatment duration from 8-9 weeks to 4-6 weeks could relieve some of the burden on the healthcare system. From the patients' perspective, a shorter treatment course would be more convenient and require a shorter absence from work.

There are multiple limitations of our study. Firstly, estimating the target population of patients receiving radiation is extremely difficult. We used data from 2014, but multiple trends may cause this estimation to be inaccurate. The US Preventive Services Task Force (USPSTF) advised against PSA screening in 2011 [26], leading to a reduction in screening and thus treatments. However these guidelines were updated in the 2017 draft, and while recommending against screening in men aged 70 or older, they recommended individual patient decisions for screening in men aged 55-69 [27]. It is expected that PSA screening and thus treatment will therefore increase. In addition, however, there has been a growing trend towards using active surveillance for low risk disease [28]. In order to perform comparisons, we regarded all patients treated with EBRT in 2014 as if they were treated with conventional fractionation, when in reality, some may have been treated with hypofractionation. Pelvic nodal irradiation is another subject we cannot account for. While most high risk trials have treated the pelvic lymph nodes, two major trials addressing this issue specifically are controversial [29, 30]. Whether elective nodal irradiation improves outcomes for high risk patients would hopefully be determined with the results of RTOG 0924. Moderate hypofractionation trials have not targeted the pelvic nodes with the exception of a small subset in one trial [17] and are not recommended by the updated guidelines for high risk patients requiring nodal irradiation [21]. It is unknown whether moderate hypofractionation could safely treat pelvic lymph nodes in case this proves to be efficacious in RTOG 0924. While most trials have found standard fractionation and moderate hypofractionation to be equally tolerated, a few have demonstrated a higher incidence of GI or GU toxicity [12, 19]. This could have an economic and clinical impact that was not considered. The recently updated ASTRO, ASCO, and AUA guidelines have concluded that while there is limited follow-up, moderate hypofractionation and standard fractionation have a similar risk of GI and GU toxicities [21]. When calculating costs, we referred to the CPT codes by CMS. When we attempted to calculate costs based on the Hospital Outpatient Prospective Payment System (OPPS), we found that access to hospital and physician specific billing was limited. While we realize that the CPT coding system represents only one aspect of the US healthcare system, its relative conformity and easy access proved more suitable for the purpose of this study. In addition, concerns have been raised that SEER data may underreport radiotherapy use. In a survey of breast cancer patients, 273 of 1292 patients who reported receiving radiotherapy were coded as not receiving radiotherapy in SEER [31]. As such, we might be underestimating the potential saving with moderate hypofractionation. Our frequency analysis of the SEER database for localized prostate cancer in 2014 found that 34,104 patients were referred for EBRT. We excluded 22,670 patients for whom EBRT status was unknown. This very large number could have significant implications on cost estimates (Figure 3, “status unknown” bar). It is likely that at least some of those patients did receive EBRT. Knowing that proportion could significantly affect the estimated total annual saving, as population size has a major impact.

Extreme hypofractionation (stereotactic body radiotherapy, SBRT) has been explored in multiple reports and in all risk groups [32–34]. Although results are encouraging, to the best of our knowledge, no randomized trial has yet to show outcome data with these regimens compared to the current standard of care. We therefore did not include SBRT in this analysis. Early toxicity results from a phase III study comparing SBRT with conventionally fractionated RT for the treatment of intermediate risk prostate cancer have been published. While there was slightly more toxicity in certain patient-reported outcome measurement in the SBRT arm at the end of RT and at 1 year, there were no significant differences compared to conventionally fractionation at the 2-year follow-up [35].

These data show that moderate hypofractionation could be economically beneficial, both directly to the healthcare system and indirectly through a shorter interruption in employment, likely without adversely impacting outcomes. However, moderate hypofractionation is still not considered as first line treatment by most cancer centers. Major shifts in the treatment paradigm may occur gradually. It is also clear that radiation oncology units and physicians would be impacted economically and may be reluctant to adopt this approach. As the detection of localized prostate cancers is expected to grow, and the use of radiotherapy to increase, we should try to reduce “beam on” time. Thus, should public and private payers reimburse moderate hypofractionation only? This issue should be seriously considered by healthcare payers.

## 5. Conclusion

Moderate hypofractionation may have the potential to save in the region of US$ 0.16-0.36 billion annually in the United States, likely without impacting survival or tolerability. Depending on insurance provider, this option may lead to lower personal financial toxicity. It would be reasonable for public and private payers to consider which type of radiation is most suited to reimbursement.

## Figures and Tables

**Table 1:** Moderate hypofractionation trials.

Trial	Design	pts randomized	Patient population (NCCN)	Fractionation and Total dose	5-year FFF^1^ rate
CHHiP	Non-inferiority	3216	73% IR^2^	37 X 2Gy, 74GY 20 X 3Gy, 60Gy 19 X 3Gy, 57Gy	88.3% 90.6% 85.9%

PROFIT	Non-inferiority	1206	100% IR	39 X 2Gy, 78Gy 20 X 3Gy, 60Gy	79% 79%

RTOG 0415	Non-inferiority	1092	100% LR^3^	41 X 1.8Gy, 73.8Gy 28 X 2.5Gy, 70Gy	85.3% 86.3%

HYPRO	Superiority	804	73% HR^4^	39 X 2Gy, 78GY 19 X 3.4Gy, 64.6Gy	77% 81%

Pollack et al.	Superiority	303	64% HR	38 X 2Gy, 76Gy 26 X 2.7GY, 70.2Gy	85% 81%

Arcangeli et al.	Superiority	168	76% HR	40 X 2Gy, 80Gy 20 X 3.1Gy, 62Gy	79% 85%

Hoffman et al	Superiority	204	70% IR	42 X 1.8Gy, 75.6GY 30 X 2.4Gy, 72Gy	92% 96%

^1^FFF: freedom from failure.

^2^IR: intermediate risk.

^3^LR: low risk.

^4^HR: high risk.

**Table 2:** CPT^1^ codes and prices, fractionation options, and costs.

**CPT Code, Description**	**Price per Unit** ^∗^	**Number of Units**
77301, IMRT^2^ treatment planning	$2033.26	1

77263, treatment planning complex	$170.64	1

77338, MLC^c^ design for IMRT	$527.39	1

77300, Basic Dose Calculation	$68.76	2

G6015, IMRT treatment delivery^∗∗^	$358.00	By number of fractions

77336, physics consult hour^∗∗^	$82.80	45 fx^4^ – 9; 39 fx – 8; 28 fx – 6; 20 fx – 4

77014, cone beam CT^∗∗^	$122.40	By number of fractions

77427, weekly treatment management^∗∗^	$191.16	45 fx^4^ – 9; 39 fx – 8; 28 fx – 6; 20 fx – 4

**Fractionation Scheme **	**Cost per Patient**	

45 fractions	$26,782	

39 fractions	$23,625	

28 fractions	$17,793	

20 fractions	$13,402	

^1^CPT: current procedural terminology.

^2^Intensity modulated radiotherapy.

^3^MLC: multileaf collimator.

^4^Fx: fractions.

^∗^The total price per code includes the professional component and technical charge when applicable.

^∗∗^Number of units varies by treatment duration.

## Data Availability

The data used to support the findings of this study are included within the article.
